# Scientific Reasoning in Biology – the Impact of Domain-General and Domain-Specific Concepts on Children’s Observation Competency

**DOI:** 10.3389/fpsyg.2020.01050

**Published:** 2020-05-26

**Authors:** Janina Klemm, Pamela Flores, Beate Sodian, Birgit J. Neuhaus

**Affiliations:** ^1^Biology Education, Faculty of Biology, Ludwig-Maximilians-Universität München, Munich, Germany; ^2^Department of Psychology and Educational Sciences, Institute for Developmental and Educational Psychology, Ludwig-Maximilians-Universität München, Munich, Germany

**Keywords:** scientific reasoning, domain-specific, domain-general, observation competency, kindergarten, biology

## Abstract

Research on the development of scientific reasoning has put the main focus on children’s experimentation skills, in particular on the control-of-variables strategy. However, there are more scientific methods than just experimentation. Observation is defined as an independent scientific method that includes not only the description of what is observed, but also all phases of the scientific inquiry, such as questioning, hypothesizing, testing, and interpreting. Previous research has shown that the quality of observations depends on specific knowledge in the domain. We argue that observation competency shares the domain-general ability to differentiate hypotheses from evidence with other scientific methods. The present study investigates the relations of both domain-general scientific thinking and domain-specific knowledge in biology with observation competency in grade K children. We tested relations between observation competency, domain-general scientific reasoning, domain-specific knowledge, and language abilities of 75 children (age 4;9 to 6;7). Both scientific reasoning and domain-specific knowledge proved to be significant predictors of observation competency, explaining 35% of the variance. In a mediation analysis, we found a significant indirect effect of language via these two predictors. Thus, the present results indicate that observation skills require not only domain-specific knowledge but also domain-general scientific reasoning abilities.

## Introduction

Scientific thinking in children, which is understood as “the application of the methods or principles of scientific inquiry to reasoning or problem-solving situations” (Zimmerman, 2007), has been primarily studied with respect to experimentation skills. Young children’s ability to design experiments and to draw valid conclusions from data has traditionally been described as severely deficient, lacking the fundamental conceptual differentiation of hypotheses from evidence (Kuhn, 1989). However, a growing body of recent research indicates that elementary school students and even kindergarteners may, in fact, be able to distinguish hypotheses from evidence and reason about the relation between the two in simple, knowledge-lean tasks. Sodian et al. (1991) showed that first- and second-graders were able to distinguish hypothesis testing from effect production and preferred a conclusive test for a simple hypothesis over an inconclusive one. Subsequent research by Piekny et al. (2013b) showed that even 44% of 5-year-olds were able to pass this task. More generally, kindergarteners seem to be able to choose adequate experiments (Leuchter et al., 2014; van der Graaf et al., 2015) and to interpret simple data sets (Koerber et al., 2005; Piekny et al., 2013a), unless when biased by prior beliefs (Koerber et al., 2005; Croker and Buchanan, 2011).

Once young children understand the inferential relation between hypotheses and evidence, they should be able to explore phenomena in the real world guided by their ideas (hypotheses) and to interpret data (observations) with respect to these hypotheses. To date, young children’s exploration skills have been mostly studied in causal learning paradigms, in which arbitrary relations between causal factors and an effect (e.g., a lightbox) had to be discovered (e.g., Gopnik and Wellman, 2012). In the present paper, we focus on kindergarteners’ exploration skills in a knowledge-rich real-world domain, the observation of animals in biology.

Observation is a key research method and an important element of science curricula (Johnston, 2009). It is relevant for social sciences (qualitative and quantitative observation of behavior) and for natural sciences, such as physics (in the field of astronomy) and biology, as it was the underlying method for Darwin’s development of the theory of evolution (Kohlhauf et al., 2011). It is important to distinguish clearly between the different meanings that are ascribed to observation in the literature. It is, on one hand, regarded as a basic process in scientific research: It is needed in all stages of an inquiry and is therefore relevant for other scientific methods; e.g., when experimenting, we need to be able to make observations in the different conditions of the design. On the other hand, it is defined as an independent and complex research method that includes not only the description of what is observed, but also all phases of the scientific inquiry, such as questioning, hypothesizing, testing, and interpreting (Kohlhauf et al., 2011; see also Oguz and Yurumezoglu, 2007; Eberbach and Crowley, 2009).

From infancy, observation is a powerful learning mechanism for children (Rogoff et al., 2003). However, little is known about the early development of scientific observation competencies, that is, the ability to systematically use observation as a tool for intentional knowledge seeking. Norris (1984) defines observation competency as the ability to make accurate observations, to report them well and to correctly assess reports of observations. Based on this conceptualization of observation competency as a specific research method, Kohlhauf et al. (2011) developed a competency model, identifying the following dimensions as important for the quality of observation: describing details, questioning, hypothesizing, testing, and interpreting. The authors describe behavior in these dimensions on three ascending levels of incidental observation, unsystematic observation, and systematic observation. In order to validate the model, they analyzed the observation behavior of 110 study participants aged between 4 and 29 years. Kindergarteners were generally on the first level (incidental observation), but even adults did not always reach the third level (systematic observation). The results confirmed a three-dimensional model: describing details, scientific reasoning (questioning, hypothesizing, and testing), and interpreting.

**FIGURE 1:**
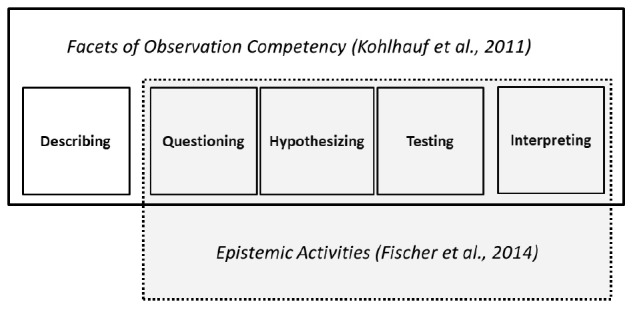
Facets of observation competency and their relation to epistemic activities.

The observation competency model by Kohlhauf et al. (2011) differentiates several important facets in observation: describing, questioning, hypothesizing, testing, and interpreting. As can be seen in Figure 1, questioning, hypothesizing, testing, and interpreting are general epistemic activities that are relevant for scientific reasoning processes across domains (Fischer et al., 2014). The cognitive and metacognitive skills needed for these processes are assumed to be domain-general (Piekny et al., 2013b). Only the description of details is specific to observation. Research on observation in the domain of biology has shown that the correct perception and description of relevant details are crucial for making good observations (Eberbach and Crowley, 2009). In this paper, we will treat describing and epistemic activities as two subscales of observation competency.

We ask whether and to what extent kindergarteners’ observation of animals, when prompted by an adult, can be described as a scientific reasoning process, characterized by questions/ideas/hypotheses, and the evaluation of observations with respect to these questions or ideas. Further, we ask whether children’s domain-independent scientific reasoning competency, as assessed in a knowledge-lean experimentation task, predicts their observation competency in biology, when domain-specific knowledge and other relevant abilities (such as language) are taken into account. Thus, the present study reflects the idea that there is not just one scientific method but several methods with their own structure and difficulties (Lederman et al., 2002). While experimentation and observation are distinct research methods, for both the relevance of both domain-general epistemological understanding and domain-specific knowledge have been shown repeatedly (Chen and Klahr, 1999; Zimmerman, 2007; Eberbach and Crowley, 2009; Kohlhauf et al., 2011).

In general, the quality of an observation has been shown to be strongly influenced by the observer’s knowledge in the domain: Observers are influenced by their prior conceptualizations in what they observe or what they think they see (Brewer and Lambert, 2001). Chinn and Malhotra (2002) showed that 75% of students did actually observe incorrectly when the correct observation was not in line with their prior conceptualization. The failure to make correct observations hindered their conceptual change. Yet, there are reasons to assume that observation competency can be a helpful tool in knowledge acquisition and conceptual change (Eberbach and Crowley, 2009). Studies comparing laypersons’ and experts’ observations show that the two groups use different strategies, with laypersons making several mistakes throughout the whole inquiry process (Eberbach and Crowley, 2009). The experts ask more specific questions and go on questioning and noticing details. Meanwhile, laypersons often ask wrong questions, miss important details, and do not document their observations adequately. Again, this can be seen during the whole inquiry process: domain knowledge is needed to ask the right questions, plan an adequate observation situation, document meaningful details and draw the right conclusions from the data (Alberdi et al., 2000). Kohlhauf et al. (2011) also found that prior knowledge of the object of investigation had a positive impact on the observation competency of their participants, who ranged from kindergarteners to university students. Since domain-specific knowledge has proved to be crucial for children’s observation competency (Eberbach and Crowley, 2009; Kohlhauf et al., 2011), we expect children’s prior knowledge about the observed objects to have an impact on the performance in the observation situation.

Since the model of observation competency proposed in the present paper places a strong emphasis on epistemic activities involved in the observation process, we further expect domain-general scientific reasoning skills to play a role in the development of observation competency. There is evidence for a development of a domain-general scientific reasoning skill (Osterhaus et al., 2015; Piekny et al., 2013a, b). Thus, we expect to find a correlation between children’s grasp of foundational epistemological distinctions and their inquiry skills in an observation situation. While many researchers in science education postulate that domain-specific knowledge is the main motor for the development of scientific skills (Sinatra and Chinn, 2012), we expect both domain-specific knowledge and domain-general understanding of hypotheses and evidence to have an impact on children’s performance in a scientific inquiry situation.

Observation competency consists of several facets (compare Figure 1). While questioning, hypothesizing, testing, and interpreting are all general epistemic activities and therefore part of all scientific reasoning processes (Fischer et al., 2014), we expect these to show a specific relation with children’s domain-general scientific reasoning. The perception and description of relevant details are expected to depend more on prior knowledge in the domain (Eberbach and Crowley, 2009).

There is little investigation of the influence of general cognitive skills on observation competency. However, it has been shown that social interaction is important for developing children’s observation skills: in the study by Johnston (2009), children observed and sorted several objects and were interviewed about their procedure. When interacting with peers or adults throughout the task, they showed and reported the use of more sophisticated strategies. Language is important for social interactions and as “intermental (social) activity will promote intramental (individual) intellectual development” (Vygotsky, 1978, p. 86), it can be expected that children with better language skills have experienced more learning situations to improve their reasoning and inquiry skills. Language has not only proved to be an important instrument in the development of false belief understanding (Lohmann and Tomasello, 2003), but also specifically for learning about science (Mercer et al., 2004). Research with children with language impairment suggests that the understanding of causal connectives is crucial for scientific reasoning (Matson and Cline, 2012). A longitudinal study showed that verbal intelligence was positively related to scientific reasoning (Bullock et al., 2009). Children’s language abilities have also been found to have an impact on both observation competency (Kohlhauf et al., 2011) and scientific reasoning (Mayer et al., 2014). Therefore, we expect an influence of language on children’s reasoning abilities, domain-specific knowledge, and observation competency. As children’s executive functions have shown to be related to their scientific reasoning skills (van der Graaf et al., 2016; Osterhaus et al., 2017), we will also measure them in order to be able to control for a potential influence on children’s performance in the tasks.

## Materials and Methods

### Sample and Procedure

Eight kindergartens were asked if they wanted to participate in the study, and five actually agreed to do so. Two of these were in an urban environment, three in a rural area. Three of the kindergartens were run by municipal authorities, one by church and one by parent initiative. All kindergartens had basic groups for the children but also group-overarching activities.

We tested 83 children who were in their kindergarten year before starting school. Eight children were excluded from the analyses because their language abilities were so low that the testing could not be run with them as it was with the other children. The cut-off for excluding them from the sample was their performance in the language test. If their results fell into the area of “special educational needs,” their performance was not analyzed any further. The age of the final sample of 75 children ranged from 4;9 to 6;7 (years;months); the mean age was 5;6 (65.56 months, SD = 4.67). A total of 38 (51%) of the children were female and 37 (49%) were male.

The testing took place in the kindergartens in a separate room. We tested the children individually in three test blocks, one testing with the observation test, one with the language test and the executive functions test, and one with the scientific reasoning tasks. Each child was usually tested on three different days; only some children were tested twice a day. In these cases, we made sure that they had at least 2 h leisure time in between. The testing either took place at a computer (language test) or was recorded on videotape. If the child did not want to be tested alone, one of the teachers would come along to the testing.

This study was carried out in accordance with the recommendations of the ethics committee of the faculty for psychology and education at the LMU Munich. As the study was conducted with minors as subjects, all parents or legal guardians gave written informed consent. They had the possibility to withdraw their consent at any time and ask for the deletion of already recorded data. The children themselves also had the possibility to cancel the testing at any time. Parents had the opportunity to ask for their own children’s test results.

### Instruments

#### Observation Competency

As mentioned in the introduction, we characterize observation competency as consisting of the two subscales describing and general epistemic activities. The latter subscale in turn consists of the facets questioning, hypothesizing, testing, and interpreting (see Figure 1).

For testing children’s observation competency, we used the procedure established by Kohlhauf et al. (2011). Here the participants observed a living fish, snail, and mouse. The instructor started the test by introducing a hand puppet and presenting the tools the children could, later on, use for their observations (magnifying glass, ruler, stopwatch, scales, and thermometer). At this point, the animal’s cages were still hidden under blankets. After that, the children were shown the first animal, which was always the fish. The puppet closed its eyes and the children were asked to describe the animal to the hand puppet. When the child had finished the description, the puppet opened its eyes again and the experimenter asked the child for a research question (“what do you want to find out about the fish?”). When they had formulated a research question (e.g., “Does the fish have to surface in order to breathe?”), the children had to generate a hypothesis (e.g., “I think the fish must come to get some air”). The children should then observe the animals and try to find answers to their own questions using various aids (e.g., the stopwatch and observe whether or not the fish had to breathe within the selected time). The last step in the observation was to sum up the observation and decide whether to accept or reject the hypothesis that has been set up (e.g., the fish has not surfaced within the set time: does it have to breathe or not?). After observing the fish, the same procedure followed with the snail and lastly the mouse. The whole interaction was videotaped.

The test was designed to find out if the participants are able to describe what they observe, come up with a research question, formulate a hypothesis, do the testing and interpret their observation. Therefore, as little prompting as possible was given by the instructor. If the child got stuck, did not do one of the steps themselves or asked for help, help or prompts were given either by the instructor or the puppet. The need for prompting resulted in scoring less points in the overall score, which will be explained in detail further below.

Table 1 displays two examples of children’s actions in the situation. While Child A needed lots of help and prompting, Child B did many steps spontaneously or only needed prompting. As these examples show, Child A did not provide a research question and needed help in order to develop the hypothesis for the given research question. Meanwhile, Child B came up with a usable research question and could form a hypothesis when prompted. In the testing phase, Child A stayed passive and the instructor both gave the idea how to test the question as well as lead the process throughout the observation. Child B, in contrast, came up with the testing idea himself/herself (looking with a magnifying glass) and executed the observation autonomously. Child A did not make a real observation before being prompted to look properly by the instructor. In the beginning of the interpretation, both children could summarize the results when prompted. While Child B also put the results into relation with the hypothesis, Child A failed to do so on his/her own. Neither of the children was able to actively separate between their observation and their interpretation. Examples for this would be any consideration of the limitations of the observation; e.g., stating that the observation would have to be repeated or that the results might be limited to the individual animal instead of being applicable to the whole species, or that the measurement might be imprecise (e.g., when trying to measure the length of the mouse through the glass of the cage).

**TABLE 1:** Examples of children’s behavior in the observation situation and coding.

**Phase**	**Child A**		**Child B**	
	**Transcript**	**Coding**	**Transcript**	**Coding**
Describing	*Instructor*: Can you describe the fish to Emil (=hand puppet)? *Child A*: A tail that is silvery and with spots on it, it has small eyes, they are black.	Dimensions: 2 Details: 4	*Instructor*: Can you describe the fish to Emil (=hand puppet)? *Child B*: The fish have orange tail fins with black dots, they are green-transparent, they have eyes and a fin on the back and side fins.	Dimensions: 5 Details: 3
Questioning	*Instructor*: Is there something you want to find out about the fish? *Child A*: No. *Instructor*: Okay, I have a question: which fins do they swim with? (Pause)	No question Instructor’s question used for investigation	*Instructor*: Is there something you want to find out about the fish? *Child B*: I want to see the very thin stems going up inside or go to the sides, I want to find out how they look (gets up and picks up the magnifying glass) *Instructor*: The fishbone? *Child B*: Yes.	Question when prompted Child’s question usable for investigation
Hypothesizing	*Instructor*: What do you think? *Child A*: With the small ones. *Instructor*: Which? Where are they? (Pause) Are they here or here (shows at own body) *Child A*: Here (shows shoulders)	Hypothesis with help	*Instructor*: And what do you think how they look like? *Child B*: Hmmm… White, and curved.	Hypothesis when prompted
Testing	*Instructor*: Okay, so let’s have a look! (Pause) Which fins do they use? *Child A*: (without really looking) These up here (shows shoulders) *Instructor*: And do other fins move as well? *Child A*: (looks) those down there *Instructor*: Those down at the belly, aha. More? *Child A*: The tail.	Idea: Mostly instructor Execution: Mostly instructor Real observation Specific details: 0	*Instructor*: Okay, so let’s have a look… you have the magnifier glasses already, but I could also catch the fish in this magnifying glass container… *Child B*: Yes! *Instructor*: (catches a fish with the help of *Child B*) *Child B*: (looking at the fish) White and curved! … and I now can see a fin on the back that I have not seen before.	Idea: Mostly child Execution: Mostly child Real observation Specific details: 2
Interpreting	*Instructor*: Okay, so what have we seen now? *Child A*: That they move those up here, those down at the belly, and the tail. *Instructor*: And what did you think before? (Pause) Do you remember? *Child A:* (no answer) *Instructor*: So you said they move only those at the shoulders. Is that right? *Child A*: No.	Summary when prompted No relation to hypothesis No separation interpretation/observation	*Instructor*: Okay, so what did you find out? *Child B*: That the fishbone are white and curved. *Instructor*: And what did you think before? *Child B*: The same. But I wanted to be sure.	Summary when prompted Relation to hypothesis on demand No separation interpretation/observation

We first analyzed children’s observations according to the procedure of Kohlhauf et al. (2011). In their analysis there were five items (one each for details, questioning, hypothesizing, testing, and interpreting) for each of the three animals, summing up to 15 items in total. This had worked well for their sample that had an age range from kindergarteners to students but proved to be too imprecise for our sample. The children showed floor effects and we were unable to reach satisfactory interrater reliability. We therefore developed a new coding scheme with more items and more gradations within each item. Our final coding scheme for each animal consisted of five facets (describing details, questioning, hypothesizing, testing, and interpreting) with a total of 13 items, which contained up to four gradations. The more autonomous and spontaneous the behavior of the child, the higher the score they were able to achieve. The list of items can be found in Table 2, including the scores the examples Child A and Child B from Table 1 got for their observation of the fish.

**TABLE 2:** List of items measuring observation competency.

**Facets**		**Items**	**Example**	
			**Child A**	**Child B**
Describing details		Dimensions	0.8	1
		Unspecific details	0.63	0.38
		Specific details	0	1
Epistemic activities	Questioning	Research question	0	0.67
		Use of question	0	1
	Hypothesizing	Spontaneous hypothesis	0	0
		Prompted hypothesis	0.67	0.67
	Testing	Activity	0	0.67
		Quality	1	1
	Interpreting	Summary of results	0.5	0.5
		Spontaneous relation to hypothesis	0	0
		Prompted relation to hypothesis	0	0.5
		Differentiation between observation and inferences	0	0

The first facet consisted of three items that focused on children’s perception of details both during their first description of the animal as well as during the testing phase. One of these three items regarded the number of dimensions (e.g., body parts and overall color) mentioned by the children. The other two items regarded the number of details (e.g., form or color of a specific body part or a description of behavior) that were mentioned by the children. With these two items, we differentiated between “specific” and “unspecific” details to distinguish whether the mentioned details were related to the research question or not. In the examples in Table 1, Child A mentioned two dimensions (tail and eyes) and two details for each (tail: silvery with spots, eyes: small and black). Child A did not relate to specific details during the testing phase (only mentioned body parts). Child B described five dimensions (tail fin, green-transparent, eyes, fin on the back, and side fins) and three details related to the tail fin (orange with black dots). As the descriptions of “white and curved” related to the research question on the fishbone, those were counted as two specific details.

The next three facets (questioning, hypothesizing, and testing) consisted of two items each. The items measured both the quality of the performance as well as whether the children performed the steps spontaneously or if they needed prompting. For this, each item contained 2–4 gradations in order to take the extent of the prompting into account.

Finally, the last facet (interpretation) included four items in which children’s summary of the results, their ability to relate them to the hypothesis and the differentiation between observation and inferences were scored. Here again, each item consisted of 2–3 gradations in order to represent the extent to which the children needed prompting. As mentioned above, neither Child A or B differentiated between their observation and their interpretation.

Since the amount of gradations differed between the items, all items were transformed to a value between 0 and 1. In the case of the first facet, in which there were no gradations, the maximal score was transformed to the value of 1 and all other scores were calculated as a percentage. In the other facets the value of 1 represented the highest gradation, e.g., the most autonomous and spontaneous behavior. Therefore, if an item consisted of three gradations, these would be ascribed with the values of 0, 0.5, and 1, respectively.

The 13 items were the same for all three animals, meaning children could reach an overall score between 0 and 39. Child A from our examples (Table 1) had an overall score of 11.3, Child B had an overall score of 20.9.

A second rater coded 10% of the data and the Spearman correlations were all above 0.6; for the facets questioning, hypothesizing, testing, and interpreting they were all above 0.9.

As mentioned earlier, we differentiate between the two subscales describing and general epistemic activities (questioning, hypothesizing, testing, and interpreting) (see Figure 1). In the analyses, we had a look at the overall scale, the two subscales describing and epistemic activities, and the facets of observation individually (describing, questioning, hypothesizing, testing, and interpreting). The overall scale of observation was reliable (α = 0.74). The subscales for details (α = 0.72) and the epistemic activities (α = 0.76) also showed satisfactory reliability. The values of the facets were only sufficient for questioning (α = 0.77) but not for the other facets (hypothesizing: α = 0.48, testing: α = 0.63, interpreting: α = 0.40). We therefore did not conduct any further inference statistics with the facets but will still report the descriptive results.

#### Scientific Reasoning

We used two tasks to measure children’s scientific reasoning abilities: the mouse task by Sodian et al. (1991) and the cake task, which was developed in parallel to the mouse task. Both tasks were administered to the children in form of a story, supported with pictures. Children could point at the pictures to answer but also had to verbally justify their answers. If the justification showed a wrong concept or no justification was given, the answer was coded as wrong. For the mouse task, there were control questions on children’s understanding of the task. If the children answered these wrong, their data were coded as missing.

*The mouse task*: in this task, the children were told the story of two boys who had a mouse in their cellar. The boys had never seen the mouse and therefore did not know if it was big or small. In the first step, they wanted to feed the mouse and had to choose one of two houses (one with a small entrance and one with a big entrance) to put cheese for the mouse in. Hereby the boys wanted to make sure that the mouse could find the food, regardless of its size. In the second step, they wanted to find out if the mouse is big or small and again had to choose one of the two houses to put cheese in. We added a third step, in which we showed the big house, saying the cheese is missing and asked the children if they now knew whether it was a big or a small mouse. With these steps, we assessed our participant’s understanding of producing an effect (first step) and of testing a hypothesis using a conclusive test (second and third steps).

*The cake task*: in this task, a mother baked a cake with two new ingredients and her three children liked the cake a lot. In the first step, the mother wanted to bake the cake again for a birthday party and the children made suggestions what she should do. Hereby the idea was to make sure that the cake tasted the same as the first time (effect production). Child A suggested to put only one of the ingredients into the new cake, child B suggested to put both ingredients into the cake (right answer), and child C suggested to bake a cake in a square form instead of a round one. In the second step, the mother wanted to find out which of the ingredients is the one to make the cake so tasty because the ingredients were rather expensive and she only wanted to have to buy one. Child A suggested to bake one cake with both ingredients and one cake without both ingredients, Child B suggested to bake one cake with the first and one cake with the second ingredient (right answer), and Child C suggested to bake one round and one square formed cake. In the third step, the family had decided to try out Child A’s suggestion and we asked the children if they now found out which ingredient makes the cake tasty. Here again, the second and third steps assessed children’s ability to test a hypothesis using a conclusive test.

As we only wanted to analyze children’s understanding of testing and not that of producing an effect, we only considered children’s answers on the second and third steps of each task, but not their answers on the first steps. Therefore, we had answers to two questions per task, one on the selection of the right answer and one on our additional *post hoc* question. Thus, children could score 0, 1, or 2 points on both scientific reasoning tasks. The frequencies of children’s scores are displayed in Table 3.

**TABLE 3:** Frequencies of scores in the Scientific Reasoning Tasks.

	**Cake task**	**Mouse task**
0	31	36
1	16	18
2	19	12

Children’s performance on the two tasks was significantly correlated (τ = 0.38, *p* < 0.01), even after language and age had been partialed out (*r* = 0.31, *p* < 0.05). Because of these correlations, we decided to aggregate the two scores to a single scientific reasoning score.

#### Prior Knowledge Test

We conducted the same test on children’s prior knowledge that Kohlhauf et al. (2011) used in their study. The questionnaire consisted of 18 questions about the three animals that were part of the observation situation. The children answered these questions verbally and their answers were written down by the experimenter. Due to floor effects, items that were solved correctly by less than two children had to be deleted. The final scale had 10 items and reached a satisfactory reliability (α = 0.58).

#### Language Abilities

We used the CITO language test (Konak et al., 2005) to measure children’s German language abilities. This is a computer-based test to evaluate children’s language abilities between age 4;3 and 6;11. The testing took about 25 min. There are four subscales in the test.

In *passive vocabulary*, the children were supposed to click on a picture that displays a word that they were asked to click on. This could either be a noun (e.g., “click on stairs”) or a verb (e.g., “click on swimming”). In *cognitive terms*, they also had to click on the right picture, but the content was more complicated. The target could be a color (e.g., “click on white”), the size of an object (e.g., “click on the tallest child”), the number (e.g., “click on the basket with the most apples”), or position of an object (e.g., “click on the house between the trees”). In *phonological awareness*, children heard either two words that sounded very similar or twice the same word. They then had to decide whether it was the same word or two different words. In *text comprehension*, the children heard a short story (4–5 sentences) and afterward were asked multiple-choice questions that tested if they had understood the story correctly and could remember the content. The reliabilities for the subscales were all sufficient to good, being as good as or even better than the ones reported by the authors (see Table 4).

**TABLE 4:** Reliabilities of the language test CITO.

**Subscale**	**Test manual**	**Our sample**
Passive vocabulary	0.91	0.89
Cognitive terms	0.88	0.89
Phonological awareness	0.79	0.88
Text comprehension	0.76	0.82
Overall		0.96

#### Executive Functions

To measure executive functions as an additional control variable, we used the Hearts & Flowers (H&F) task, a computerized test, developed by Diamond (2013). The test was constructed to assess inhibition, set-shifting, and working memory (Davidson et al., 2006; Diamond et al., 2007). The test items were displayed on a computer screen, on which items could be seen on either the left and the right side, and a keyboard with an active key on the left and an active key on the right side. There were three conditions: congruent, incongruent, and mixed. In the congruent condition, children were asked to click the key on the same side as the heart appears. In the incongruent condition, children were asked to click on the opposite side of the flower that appears on the screen. In the mixed condition, either a heart or a flower could appear. If it was a heart, children had to click on the same side; if it was a flower, they had to click on the opposite side. The congruent and incongruent condition each contained 20 trials, the mixed condition contained 33 items.

In the mixed condition, children had to keep both rules in mind (working memory), shift between the rules and inhibit the tendency to press the key on the same side in incongruent items. This condition was therefore the best measure for executive functions and demanding enough to not produce ceiling effects (Zaitchik et al., 2014). Consequently, we used only this scale for our analyses. It reached good reliability (α = 0.85).

#### Statistical Analysis

For the data analysis, we conducted descriptive statistics and calculated correlations and multiple regression analysis using the software IBM SPSS Statistics for Windows, version 24.0. Furthermore, we conducted a mediation analysis using the software PROCESS by Hayes (2012). This program does not only offer to run the Sobel test to determine if the mediation is significant but also gives out bootstrap confidence intervals.

## Results

### Descriptive Results

The first aim of the analyses was to assess the level of children’s observation competency. Additionally to the analysis of the overall observation competency scale, we also had a look at the subscales and facets. For this, we considered describing and the epistemic activities as subscales, as well as all the other facets (questioning, hypothesizing, testing, and interpreting) individually. Table 5 displays descriptive for all subscales/facets.

**TABLE 5:** Descriptive for overall observation competency, subscales, and facets.

**Variable**	***M***	***SD***
Observation competency (sum score)	15.19	4.13
Epistemic activities	0.41	0.11
Describing	0.40	0.15
Questioning	0.43	0.30
Hypothesizing	0.48	0.20
Testing	0.70	0.19
Interpreting	0.20	0.10
		

In the original study by Kohlhauf (2013), the participants had been sorted into three levels of observation competency. With this categorization, children of that study showed floor effects, as kindergarteners were mostly on the lowest level. Given that, this study could not use the same coding scheme and therefore, the categorization could not be applied in the same way. However, children’s performance in the test could still be differentiated in their solving rates of the tasks. For this, we chose to divide the participants in three levels: On level 0, participants scored less than 20% of the points given in the subscale. On level 1, they solved between 20 and 80% of the task. On the highest level (level 2), they solved over 80% of the task correctly. This way, we could better recognize the variability in our sample while examining whether our coding scheme would still elicit floor effects. Table 6 shows the distribution of the children across the three levels. For the subscales describing and hypothesizing, almost all children were on the medium level. For questioning and testing, the children showed a broader distribution, with testing being the facet with most children in the highest level. Interpreting showed half the children on the lowest and half the children on the medium level.

**TABLE 6:** Descriptive for observation competency levels.

**Facet**	**Level 0 (<20%)**	**Level 1 (20–80%)**	**Level 2 (>80%)**
Describing	8%	92%	0%
Questioning	24%	59%	17%
Hypothesizing	5%	92%	3%
Testing	3%	68%	29%
Interpreting	49%	51%	0%

### Intercorrelations With Cognitive Measures

Table 7 shows the means, standard deviations, and intercorrelations for the overall observation competency measure and the cognitive measures. The expected correlations of observation competency with scientific reasoning, prior knowledge, and language were significant and moderate to strong. Executive functions did not correlate with observation competency. Age did also not have a significant influence on any of the variables except for prior knowledge. Analyses showed that there is no influence by children’s gender on the results in any of our measurements.

**TABLE 7:** Means, standard deviations, and intercorrelations for observation competency and all predictor variables.

**Variable**	**M**	**SD**	**1**	**2**	**3**	**4**	**5**	**6**
1. Observation competency	15.19	4.13	1					
2. Scientific reasoning	0.37	0.32	0.51**	1				
3. Prior knowledge	0.13	0.15	0.44**	0.42**	1			
4. Language (vocabulary)	0.91	0.09	0.41**	0.47**	0.37**	1		
5. Executive functions	0.70	0.19	0.16	0.50**	0.17	0.45**	1	
6. Age (months)	65.56	4.67	0.20	0.21	0.31**	0.10	–0.06	1

**Significant on 1%-level.

The intercorrelations of the facets and their correlations with the cognitive measures are displayed in Table 8. Both the overall observation competency as well as the two subscales show a moderate positive correlation with language abilities, scientific reasoning, and prior knowledge. There is no significant correlation with executive functions.

**TABLE 8:** Intercorrelations among subscales and correlations with cognitive measures.

	**Observation**	**Epistemic activities**	**Describing**	**Questioning**	**Hypothesizing**	**Testing**	**Interpreting**
Observation	1						
Epistemic Activities	0.95**	1					
Describing	0.70**	0.47**	1				
Questioning	0.72**	0.74**	0.34**	1			
Hypothesizing	0.41**	0.44**	0.23*	0.04	1		
Testing	0.52**	0.59**	0.22	0.22	0.05	1	
Interpreting	0.69**	0.69**	0.40**	0.32**	0.15	0.39**	1
Scientific reasoning	0.51**	0.48**	0.38**	0.31**	0.11	0.36**	0.48**
Prior knowledge	0.44**	0.37**	0.45**	0.33**	0.10	0.27*	0.30**
Language	0.41**	0.36**	0.28*	0.15	0.32**	0.18	0.36**
Executive functions	0.16	0.17	0.05	0.16	0.12	0.01	0.18

*Significant on 5%-level; **Significant on 1%-level.

### Predicting Observation Competency

To further investigate the relations between observation competency and potential influencing factors, we used the significantly correlated variables – scientific reasoning, prior knowledge, and language – as predictors in a multiple regression analysis. All predictors together explained 35% of the variance (*R*^2^ = 0.35, *p* < 0.001). The results in Table 9 show that prior knowledge was the largest influencing factor, followed by domain-general scientific reasoning. Language abilities were not a significant predictor.

**TABLE 9:** Regression analysis summary for scientific reasoning, prior knowledge, and language predicting observation competency.

**Variable**	***B***	***SE B***	**β**	***t***	***p***
(Constant)	5.78	4.41		1.31	0.20
Scientific reasoning	4.13	1.44	0.33	2.88	0.00
Prior knowledge	6.98	3.02	0.25	2.31	0.02
Language	7.60	5.12	0.17	1.48	0.14

Language abilities were also correlated with scientific reasoning and prior knowledge (see Table 7), so one assumption is that scientific reasoning and biology understanding mediate the influence of language abilities on observation competency. In order to check for this, we conducted a mediation analysis, using the software PROCESS by Hayes (2012).

**FIGURE 2:**
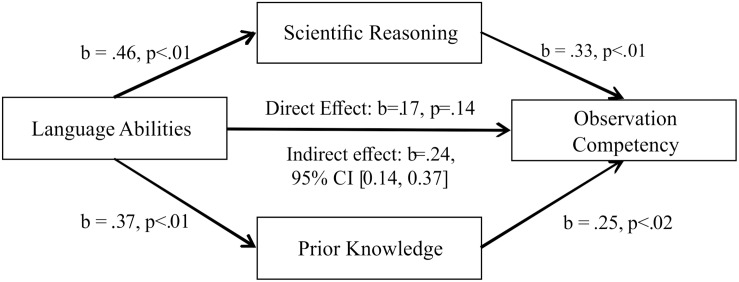
Mediation analysis of the indirect effect of language on observation competency through scientific reasoning and prior knowledge.

We used observation competency as the criterion, language as the independent variable and scientific reasoning and prior knowledge as mediators. There was a significant indirect effect of language on observation competency through both predictors [*b* = 0.244, BCa CI (0.235, 0.365)], as well as a significant indirect effect through only scientific reasoning [*b* = 0.153, BCa CI (0.046, 0.268)], and through only prior knowledge [*b* = 0.093, BCa CI (0.019, 0.180)]. The Sobel test was significant for scientific reasoning (*p* = 0.017), but not for prior knowledge (*p* = 0.064). The results are displayed in Figure 2.

### Predicting Subscales of Observation Competency

Expecting a specific relation between the general epistemic activities (questioning, hypothesizing, testing, and interpreting) and domain-general scientific reasoning on the one hand and between describing details and prior knowledge, two further regressions were conducted to check these relations. Children’s language abilities were kept as a control variable.

Domain-general scientific reasoning, prior knowledge, and language abilities explained 25% of the variance in children’s describing (*R*^2^ = 0.25, *p* < 0.001) and 28% of the variance in children’s performance throughout the epistemic activities (*R*^2^ = 0.28, *p* < 0.001). For both scales, language abilities were not a significant predictor (see Tables 10, 11). While prior knowledge was the only significant predictor for describing, scientific reasoning was the only significant predictor for epistemic activities.

**TABLE 10:** Regression analysis summary for scientific reasoning, prior knowledge, and language predicting general epistemic activities in observation competency.

**Variable**	***B***	***SE B***	**β**	***t***	***p***
(Constant)	0.18	0.13		1.44	0.16
Scientific reasoning	0.12	0.04	0.33	2.78	0.01
Prior knowledge	0.14	0.09	0.19	1.64	0.11
Language	0.18	0.15	0.14	1.20	0.23

**TABLE 11:** Regression analysis summary for scientific reasoning, prior knowledge, and language predicting describing in observation competency.

**Variable**	***B***	***SE B***	**β**	***t***	***p***
(Constant)	0.24	0.17		1.45	0.15
Scientific reasoning	0.10	0.06	0.22	1.77	0.08
Prior knowledge	0.34	0.12	0.34	2.90	0.01
Language	0.09	0.20	0.05	0.44	0.67

## Discussion

The present study is the first systematic investigation of scientific observation competency in young children. Observation competency was defined as comprising the ability to describe features of target animals, as well as to generate questions and hypotheses with regard to the target animals, to test these hypotheses, and to interpret the findings with respect to the question or hypothesis (epistemic activities). Our first aim was to describe the scope and limits of observation competency in kindergarteners, while the second aim was to relate individual differences in children’s observation competencies to general cognitive abilities (e.g., language and executive functions), domain-general scientific reasoning skills and domain-specific knowledge.

The descriptive data indicated that there were no floor effects in kindergarteners for most facets of observation competency. One exception was the ability to generate interpretations for their observations: on this facet, about half of the sample did not respond even when prompted. Most children showed evidence – at least when prompted – for some epistemic activities. It is possible (and remains to be explored further) that the differentiation of data and interpretation is harder to grasp for young children than the basic idea of testing hypotheses through specific observations.

Given that there was both a sufficient level of performance and individual variability with respect to observation competency in the present sample, it was possible to investigate the predictors of observation competency in kindergarteners.

Our hypothesis was that both children’s domain knowledge and their domain-general scientific reasoning ability would have an effect on their observation competency even if more general cognitive abilities were controlled for. These hypotheses were corroborated by the data: scientific reasoning proved to be a significant predictor for children’s observation competency alongside with children’s prior knowledge about animals. The results thus indicated that not only domain-specific competencies are important for scientific observation, but also domain-general reasoning abilities.

With respect to the effects of domain knowledge, the present findings once again demonstrate that “if it is true that thinking and reasoning are processes, so too it is true that content knowledge is the fodder for these processes” (Sinatra and Chinn, 2012, p. 258). Content knowledge was assessed as simple factual knowledge about animals in the present study. Therefore, a linear positive relation between the amount of content knowledge and the ability to describe concrete observations in animals was expected. Our findings are not inconsistent with more complex models of the interaction between children’s content knowledge and reasoning or observation skills which emphasize that prior domain-specific beliefs can be an impediment to the coordination of theory and evidence (Koerber et al., 2005; Croker and Buchanan, 2011). Research has also shown that evidence contradicting children’s prior beliefs can also lead to further and deeper inquiry (Legare et al., 2010). Further research is necessary to determine the effects of children’s conceptual understanding in the domain of biology (e.g., childhood animism and concept of living things) on young children’s observation competencies. Children’s naïve concepts of living things (Gelman, 2009) and their tendency to focus on goal-direction (Evans, 2008) would be factors that could hamper children’s observation competency, while more sophisticated knowledge about the domain may lead to better, unbiased reasoning (Geary, 2008).

The finding that the ability to distinguish between a conclusive and an inconclusive test for a simple hypothesis in an everyday domain predicted children’s observation competency in biology was predicted on the grounds that the differentiation of hypotheses from evidence is assumed to be fundamental for scientific reasoning in general, not just for experimentation skills. It should be noted that the observation competency assessment did not include the notion of a conclusive test and was not similar in terms of task demands to the scientific reasoning task. Thus, it appears that hypothesis – evidence differentiation is a metaconceptual distinction that underlies a wide range of scientific reasoning abilities, and that is domain-independent. The finding that the score attained for the general epistemic activities, not for the description of specific details, was related to children’s domain-general scientific reasoning also supports this interpretation. The perception and description of relevant details, on the other hand, was more closely related to children’s domain-specific knowledge. Eberbach and Crowley (2009) argue that laypersons with scarce domain-specific knowledge often miss the meaningful details or concentrate on irrelevant properties of the observed object.

Furthermore, we found a correlation between scientific reasoning and executive functions. This result is consistent with a growing body of findings indicating an association of scientific reasoning and executive function measures in different age groups (Mayer et al., 2014; Osterhaus et al., 2017). However, executive functions showed no relation to observation competency or any of its subscales, at least with the executive functions task used in the study. This could suggest that it is elicited reasoning, rather than spontaneous response tasks that show higher executive demands, as children’s spontaneous reactions were recorded before prompts were given. Observation competency was linked to scientific reasoning independently of executive functions, thus again supporting the idea that the metaconceptual understanding of the hypothesis evidence relation is foundational for a wide range of scientific activities. Still, other executive functions tasks with a higher emphasis on working memory or planning abilities might show a direct relation to observation. Further research is needed to better understand this relation.

We also assumed that children’s language abilities have an influence on all of the other measures. While we did find correlations between language and reasoning abilities, domain knowledge and observation competency, language ability was not a significant predictor for observation competency when we did a regression analysis with all three predictors. Our mediation analysis showed that the influence of language on children’s performance in the observation task was mediated by both domain-general and domain-specific science skills. This finding appears to be consistent with the interpretation that children’s language abilities influence both general reasoning abilities and knowledge, which both contribute to children’s abilities in a concrete observation situation. This also means that the impact scientific reasoning and prior knowledge have on the observation competency is more than just the shared influence of language: they both had a specific, independent effect on children’s performance in the observation task. Sociocultural theories postulate that intellectual competencies are a cultural product and are therefore derived through social interaction (Vygotsky, 1978; Tomasello, 2013). Our results could fortify these theories – language was an influencing factor on all our measurements. However, language seems to have a more direct influence on knowledge and reasoning, while these then shape the behavior in the scientific inquiry situation. Of course, it is also possible that the effect of language we found was a testing effect – as all our instruments were, of course, language-based, we cannot refute this alternative explanation. In this case, however, the influence of both knowledge and reasoning is more than an effect of the verbal testing method because their relation to observation competency stays significant when controlling for language.

In sum, the present study has shown that both domain-specific knowledge and domain-general scientific reasoning abilities contribute to children’s observation competency in the domain of biology. This is notable since metaconceptual foundations of scientific reasoning only begin to develop in this age group. Further research is needed to determine the interrelations of these core components of scientific activities over a wider age range. The study focused on scientific observation. Many aspects of observation competency as defined in this study are general epistemic activities, such as hypothesizing and interpreting observations. Further research is needed to determine the generalizability of the present findings to other scientific methods.

## Data Availability Statement

The dataset for this study can be found on OSF: https://osf.io/tszay/ (Klemm et al., 2020). The raw data (video recordings) cannot be made publicly available due to the protection of privacy of the study participants. Further materials have also been published in the appendices of the dissertation of Janina Klemm, “Biological Observation Competency in Preschool: the Relation to Scientific Reasoning and Opportunities for Intervention” (Klemm, 2017), accessible on https://edoc.ub.uni-muenchen.de/. The instruments used in this study can be made available to interested researchers upon request.

## Ethics Statement

The studies involving human participants were reviewed and approved by the Ethics committee of the faculty for psychology and education at the LMU, Munich. Written informed consent to participate in this study was provided by the participants’ legal guardian/next of kin.

## Author Contributions

JK, BN, and BS contributed to the conception and design of the study. JK organized the database, performed the statistical analysis, and wrote the first draft of the manuscript. All authors wrote sections of the manuscript, contributed to manuscript revision, read, and approved the submitted version.

## Conflict of Interest

The authors declare that the research was conducted in the absence of any commercial or financial relationships that could be construed as a potential conflict of interest.
